# ”Obturation capacity of lateral canals using Thermafil® with AH Plus® and a single-cone with AH Plus Bioceramic® sealer: An in vitro study”

**DOI:** 10.4317/jced.63381

**Published:** 2025-12-30

**Authors:** Alberto Albero-Monteagudo, Giangiacomo Davo, Nicolás Collado-Castellanos, María Grau-Benitez, Ángel Del Campo, Pedro Micó-Muñoz

**Affiliations:** 1 Universidad Europea de Valencia. Faculty of Health Sciences. Department of Dentistry; 2 Graduated Dentist at Universidad Europea de Valencia. Spain

## Abstract

**Background:**

Lateral root canals, which often branch from the main root canal system, contain pulp tissue and may harbor pathological processes requiring endodontic intervetion. Effective identification, cleaning, and obturation of these lateral canals are crucial for the long-term success of root canal treatment. This study compares two commonly used obturation techniques: the single-cone technique using gutta-percha with AH Plus Bioceramic® sealer, and the Thermafil® technique with AH Plus® sealer.

**Material and Methods:**

Standardized training blocks containing artificial root canal and two lateral canals (coronal and apical) were used. All canals were prepared using ProTaper Next® rotary files. Half of the samples were obturated using the single-cone technique with AH Plus Bioceramic® sealer, and the other half with the Thermafil® technique using AH Plus® sealer. Radiographs and photographs were obtained to evaluated whether lateral canals were filled, partially filled, or unfilled, and to identify the material responsible for the filling.

**Results:**

Radiographic evaluation demonstrated successful obturation in 90% of coronal lateral canals and 56.7% of apical lateral canals, resulting in an overall success rate of 73%. No statistically significant differences were observed between the single-cone and Thermafil® techniques for either coronal or apical lateral canals (p &gt; 0.05). Photographic assessment showed a non-significant trend toward improved apical filling with Thermafil® (p = 0.063). Coronal canals were significantly more likely to be filled than apical ones (p &lt; 0.001). The presence of gutta-percha combined with sealer significantly increased the filling rate compared to sealer alone, particularly in apical canals treated with Thermafil® (p &lt; 0.001).

**Conclusions:**

Although no significant differences were observed between techniques, Thermafil® exhibited a trend toward improved apical filling. Overall, coronal lateral canals were more successfully filled than apical canals, and the combination of gutta-percha and sealer was more effective than sealer alone.

## Introduction

Endodontics is the procedure for intervention in pathological processes of the pulp (1). For a favorable prognosis, it is essential that the root canal filling is hermetic and free of gaps in all areas of the canal system, including the lateral canals, as well as the periapical and coronal areas (2). Knowledge of the root canal anatomy is essential for proper access, thorough treatment of all irregularities, and effective disinfection (3). It is now considered more appropriate to refer to the root canal as a system: in addition to the main canals, accessories and lateral canals are present. The incidence of lateral canals in root canals ranges from 27% to 99% (4). These canals connect the main canal and the periodontal ligament. Although smaller in size, lateral canals may contain infected or necrotic tissue, and inadequate disinfection or filling can lead to endodontic treatment failure (5 , 6). In general, gutta-percha in combination with an intercanal sealer is considered the material of choice for root canal obturation (7). Gutta-percha provides bulk and fills the canal lumen, while the sealer adheres to the canal walls and seals apical, coronal, and, importantly, lateral canal spaces (2). The most used obturation techniques include cold lateral condensation, the single-cone technique, carrier-based techniques, and warm vertical condensation (8 , 9). The single-cone technique involves the use of a single gutta-percha cone matching the shape and size of the last rotary file used. It is considered a reliable method due to its ease of learning and application, and it provides favorable outcomes in terms of material homogeneity and apical sealing. The sealer plays a crucial role in this technique, as it is responsible for filling the remaining canal space and all anatomical irregularities (9). Thermafil® technique involves the use of a radiopaque plastic carrier wrap with a thin layer of gutta-percha. When heated in a specialized oven, the gutta-percha becomes plasticized, making the technique particularly effective for canals that are highly curved or otherwise difficult to fill (10). The main objective of this study was to determine which obturation technique is most effective in filling the greatest number of lateral canals. The secondary objectives included evaluating whether the obturation rate differs between coronal and apical lateral canals, and to observe if the lateral canals were obturated with gutta-percha, sealer or both.

## Material and Methods

**Figure 1:**
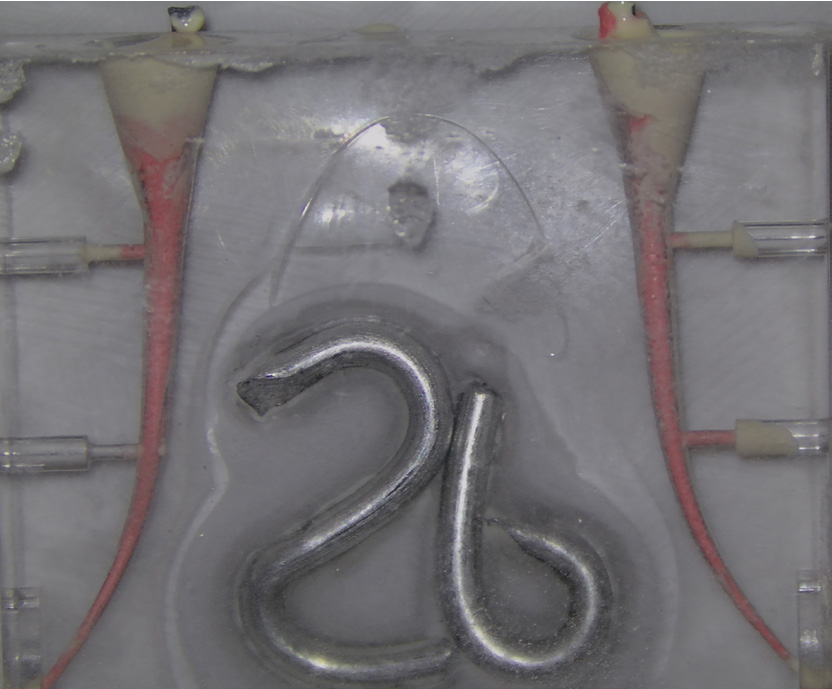
Example of the evaluation method; A26 and 26B canals, the apical canal of the A26B was considered unfilled. Apical canal of A26A was filled with gutta-percha. Coronal canals of A26A and A26B were filled with gutta-percha and sealer.

**Figure 2:**
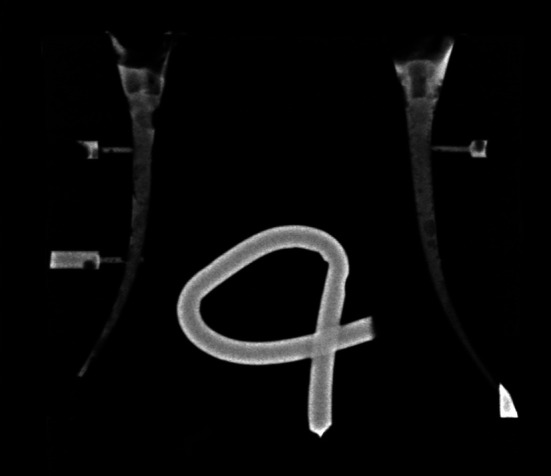
Example of evaluation method; x-ray of A4 and 4B canals, apical lateral canal 4B was considered unfilled.

The study was designed as a descriptive and analytical in vitro experimental model, following the recommendations of the ARRIVE and CONSORT guidelines adapted for in vitro study. Ethical approval was obtained from the Ethics Committee of Universidad Europea de Valencia on January 12, 2024, in Villaviciosa de Odón, Madrid, under the internal code 2024-434. Sample selection The sample was selected according to the following criteria: Inclusion criteria: methacrylate blocks with moderately curved root canals, standardized with two lateral canals connected. Exclusion criteria: absence of patency between the main canal and the apex or lateral canals due to manufacturing or operator mistakes. The final sample consisted of 60 methacrylate root canal reproductions, each with two lateral canals (30 blocks for each obturation technique). - Step 1: Nomenclature, instrumentation and patency checking Nomenclature: Two metal numbers were attached to each acrylic platform, one on each side. The canal located to the left of each number was labeled with the letter "A," and the canal to the right with the letter "B." Thus, for the first platform, the canals were designated as A1, 1B, A2, and 2B. Instrumentation: The apical constriction was first located using a size 10 K-file, and the working length was determined. Mechanical instrumentation was then performed with ProTaper Next® rotary files up to size X2, following the manufacturer's instructions. Patency checking: Patency was assessed using a syringe with water. First, both lateral canals were manually sealed to verify apical patency. The apex was then manually sealed to assess patency of the most apical lateral canal. Finally, the apical foramen and apical lateral canals were were manually closed to check the patency of the coronal lateral canals. In lateral canals where water flow was not observed, a size 10 K-file was used to confirm patency and communication with the main root canal. - Step 2: Canal filling. Half of the canals (A1 to 15B) were obturated using the single-cone technique with AH Plus Bioceramic® sealer (Procedure 1). The remaining canals (A16 to 30B) were obturated with the Thermafil® technique using AH Plus® sealer (Procedure 2). During instrumentation, two X2 files fractured in canals A11 and 24B, one X1 file fractured in canal A29, and canal A27 presented a manufacturing defect. As a result, canals Aextra1, extra1B, and Aextra2 were prepared and obturated using the Thermafil® technique with AH Plus® sealer, while canal extra2B was prepared and obturated with the single-cone technique and AH Plus Bioceramic® sealer. Procedure 1: Single-Cone Technique with AH Plus Bioceramic® Sealer The AH Plus Bioceramic® sealer was supplied in a syringe with a size 24 needle. After verifying the canal's patency and fluidity, the sealer was introduced into the root canal until the middle third was reached. A gutta-percha cone matching the size of the last file used (in this case, the red cone) was then inserted into the canal. Finally, the gutta-percha was cut with a heated instrument and compacted with a plugger. All procedures were carried out according to the manufacturer's instructions. Procedure 2: Thermafil® Technique with AH Plus® Sealer The AH Plus® sealer was placed into the root canal using paper points. The working length was established with a rubber stop, and the Thermafil® obturator was heated in the Thermaprep 2® oven before insertion into the root canal. Once the material had set, the excess coronal portion of the carrier was removed with scissors. All procedures were performed according to the manufacturer's instructions. - Step 3: Setting of the sealer To simulate oral conditions, all obturated canals were stored in a humid environment at 37 ° C in an incubator, with a glass of water placed inside to maintain moisture. The specimens were kept under these conditions for four days to allow complete setting of the sealer. Step 4: Data collection For each canal, a frontal photograph and a radiograph were obtained, using the metal number placed between the two canals as a reference marker. Photographs were taken with a ZUMAX OMS 2380 microscope at 10× magnification. The evaluation of the obturated canals was performed according to the following nomenclature: Radiographic Evaluation Lateral canals were classified as follows: 1: Totally obturated (no voids); 2: Partially obturated (presence of voids); 0: Non-obturated (no material present in the lateral canal) Photographic Evaluation Canals were classified as follows: 1: Completely filled; 2: Partially filled; 0: Unfilled For qualitative analysis of photographic data, the following variables were recorded: X: Filled with gutta-percha; Y: Filled with sealer; Z: Filled with both gutta-percha and sealer - Statistical Analysis Statistical analysis was performed using Fisher's exact test, the Chi-square test, and McNemar's test to compare the filling rates of lateral canals in radiographs and photographs, as well as to assess the influence of obturation technique, canal position, and material type (Figs. 1,2).

1

2

## Results

**Figure 3:**
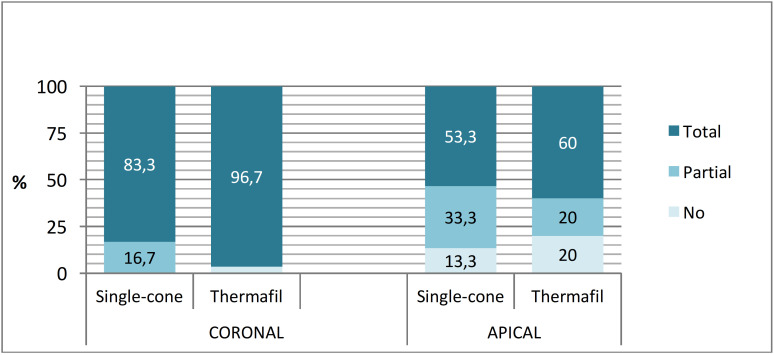
Graphical representation of the results obtained from radiographs.

- Success in the filling of lateral canals in radiographs (Fig. 3, Table 1).

3

Table 1

Overall, 90% of coronal lateral canals and 56.7% of apical lateral canals were completely filled, yielding an absolute success rate of 73% for all lateral canals. At the coronal level, 83.3% of lateral canals in the single-cone group were completely filled, compared with 96.7% in the Thermafil® group. This difference was not statistically significant (p = 0.195, Fisher's exact test). At the apical level, 53.3% of lateral canals in the single-cone group were completely filled, compared with 60% in the Thermafil® group. No statistically significant difference was observed (p = 0.602, Chi-square test). - Success in the filling of lateral canals: comparison between radiographic and photographic assessment Coronal Lateral Canal Regardless of the technique used, the total filling rate with radiographs is 90% and with photographs is 85%. There is no significant difference (p=0.375, McNemar's test). Group single-cone, the rates were 83.3% and 73.3%, respectively (p=0.375, McNemar). Group Thermafil®, the rates were 96.7% and 96.7%, respectively (p=1, McNemar). Apical Lateral Canal Across all canals, the overall filling rate was 56.7% for both radiographs and photographs, indicating absolute equivalence (p = 1, McNemar's test). In the single-cone group, filling rates were 53.3% on radiographs and 36.7% on photographs; this difference was not statistically significant (p = 0.125, McNemar's test). In the Thermafil® group, filling rates were 60% on radiographs and 76.7% on photographs, showing a non-significant trend toward improved photographic detection (p = 0.063, McNemar's test). - Influence of the position of the lateral canal (Fig. 3, Table 1) Regardless of the technique used (i.e., for all canals), the total filling rate was 90% for coronal canals and 56.7% for apical canals, a difference that reached statistical significance (p &lt; 0.001, McNemar's test). In the single-cone group, filling rates were 83.3% for coronal canals and 53.3% for apical canals, with the difference being statistically significant (p = 0.022, McNemar's test). In the Thermafil® group, filling rates were 96.7% for coronal canals and 60% for apical canals, also showing a statistically significant difference (p = 0.001, McNemar's test). - Evaluation of the type of material used to fill the lateral canal In the Thermafil® technique: Coronal lateral canals: 90% were filled with both gutta-percha and sealer, 7% with gutta-percha alone, and 3% with sealer alone. In the lateral apical canals: 71% were filled with gutta-percha alone, 25% with both gutta-percha and sealer, and 4% with sealer alone. Single-cone technique: As expected, only sealer was identified in lateral canals, whether they were completely or partially filled, and independent of canal location (coronal or apical).

## Discussion

As demonstrated in the results, both techniques were capable of filling lateral canals at the coronal and apical levels, with no statistically significant differences between them. - Successful obturation of lateral canals. Previous studies have reported that the single-cone (SC) technique-although simpler and less operator-dependent-can achieve favorable outcomes when combined with bioceramic sealers. This technique benefits from the thixotropy and flowability of bioceramic sealers, which allow penetration into areas that may be inaccessible with other methods. Chybowski et al. (2022) observed that the bioceramic sealer EndoSequence BC Sealer, when used with the SC technique, achieved penetration levels comparable to those obtained with the continuous wave of condensation (CWC) technique in simulated canals. However, its performance was inferior in terms of obturation density and resistance to apical leakage (11). In contrast, warm gutta-percha techniques offer several advantages, such as the absence of voids, greater homogeneity of the canal filling, improved adhesion to dentinal walls, and the ability to penetrate dentinal tubules, apical isthmuses, and lateral canals. Owing to these properties, they are generally considered superior to the SC technique (12). Juha et al. (13) reported a 95% obturation rate of lateral canals located 3 mm from the apex when using thermoplasticized gutta-percha in combination with BC Sealer HiFlow. In contrast, only a 38% rate was observed when the single-cone technique was employed with a conventional epoxy-based sealer. This marked difference may be attributed not only to the thermoplastic properties of gutta-percha, which enhance its adaptation to canal morphology, but also to the ability of the bioceramic sealer to flow and penetrate into irregular spaces, owing to its low initial viscosity and subsequent hygroscopic expansion. A recent study reported a lateral canal obturation rate of 55% in 360 lateral canals prepared in 70 human single-rooted teeth, whereas in the present study, radiographic analysis showed a 73% success rate (14). This difference may be explained by the type of sample used: while the previous study employed natural teeth, the present investigation used standardized methacrylate canals treated with similar materials and techniques. - Influence on the position of the lateral canal Across all groups, apical lateral canals exhibited a lower filling rate compared to those located more coronally. This finding suggests that the effectiveness of obturation techniques may be influenced by the anatomical position of the lateral canal. Canals situated in the coronal and middle thirds were more frequently and consistently filled, regardless of the technique employed, likely due to their greater accessibility. By contrast, in the apical third-where the canal anatomy is narrower and more complex-the continuous wave of condensation (CWC) technique has shown superior performance in previous studies, supporting its recommended use in cases with suspected anatomical complexity (15). On the other hand, Goldberg et al. (16) reported that although the number of obturated lateral canals was slightly higher in the coronal and middle thirds (80.83%) compared to the apical third (70%), this difference was not statistically significant among Thermafil® techniques. This contrasts with our findings, which demonstrate a clear superiority in coronal obturation. Similarly, in oval-shaped canals obturated with thermoplasticized techniques (Thermafil® and System B) or single-cone technique showed that the number of voids in the cervical third was significantly higher than in the middle and apical thirds (17). Taken together, these findings can suggest that the choice of obturation technique should be based on root canal anatomy, operator experience, and the type of sealer used. While thermoplastic techniques required specific equipment and greater clinical skill, they allowed for superior adaptation in anatomically challenging areas. Conversely, techniques such as the hydraulic single-cone may be appropriate in simple cases or in patients with lower tolerance for lengthy procedures, provided that materials are used which compensate for their mechanical limitations (18). These findings highlight the importance of individualized endodontic treatment planning, grounded in a thorough understanding of root canal anatomy, the judicious use of available technology, and adherence to the most current scientific evidence. Careful selection and proper execution of the obturation technique-particularly in teeth with complex morphologies-remain critical to achieve long-term treatment success and minimizing the risk of reinfection. - Sealer resorption In the single-cone group, it was observed that the AH Plus Bioceramic® sealer showed changes after only four days in the incubator. Grossman described the properties of an ideal endodontic sealer, one of which is long-term insolubility, a criterion that was not fully met in this case (19). It is important to note that the experimental conditions, while realistic in terms of humidity and temperature, lacked the biological components-such as organic and enzymatic factors-present in the periapical and periodontal environment. Although these limitations may suggest a potential for increased sealer resorption in vivo, further experimental confirmation is required. Otherwise, however, it is precisely the ability to bond with hydroxyapatite and induce root dentine formation that makes bioceramic sealers interesting, and that the resorption so evident in this study may be due to the material of the canals themselves, methacrylate, and not to the sealer itself. In other words, the lack of biological substrate in the canals, useful for standardizing conditions, may be a bias for the specific conclusions of this group (12). This phenomenon was not observed under the same conditions in the AH Plus® and Thermafil® groups. Additionally, the lateral canals in the Thermafil® group were also filled with gutta-percha, which is insoluble and thus may provide more durable sealing. Similarly, the epoxy-based AH Plus® sealer is insoluble, contributing to long-term stability. A recent study examined the physico-mechanical properties of different bioceramic sealers and reported that AH Plus Bioceramic®, when compared with the resin-based AH Plus® sealer, exhibited resorption in water and in a medium simulating the periapical environment, consistent with the observations in the present study (19). The amount of resorption was excessive relative to the standards it is expected to meet, thereby confirming the reliability of the present findings. - Limitations of the study It is challenging to establish comprehensive comparisons, as few studies employ the same techniques in combination with identical sealers. In this regard, the present study may be of particular interest, as it minimized manufacturer-related bias: both the filling materials and the standardized root canal models originated from the same manufacturer. - Proposals for further studies Future research should aim to determine which techniques and materials achieve the most favorable outcomes in the obturation of lateral canals, particularly in the apical third. In addition, longitudinal clinical studies assessing the long-term persistence of bioceramic sealers within lateral canals are warranted, as they would incorporate the complexity of the biological system and provide more clinically relevant evidence.

## Conclusions

Both techniques-Thermafil® with AH Plus® and single-cone with AH Plus Bioceramic®-achieved obturation of lateral canals at both coronal and apical levels, with no statistically significant differences. In general, coronal lateral canals showed a higher obturation rate than apical canals, regardless of the technique used. In the Thermafil® with AH Plus® group, lateral canals were predominantly filled with both sealer and gutta-percha in the coronal third, and mainly with gutta-percha alone in the apical third. In contrast, in the single-cone with AH Plus Bioceramic® group, lateral canals were consistently filled with sealer only.

## Figures and Tables

**Table 1:** Representation of the results obtained from radiographs.

		Technique					
		Total		Single-cone		Thermfil	
		N	%	N	%	N	%
Lateral coronal	Total	60	100,0%	30	100,0%	30	100,0%
	No	1	1,7%	0	,0%	1	3,3%
	Partial	5	8,3%	5	16,7%	0	,0%
	Total	54	90,0%	25	83,3%	29	96,7%
Lateral apical	Total	60	100,0%	30	100,0%	30	100,0%
	No	10	16,7%	4	13,3%	6	20,0%
	Partial	16	26,7%	10	33,3%	6	20,0%
	Total	34	56,7%	16	53,3%	18	60,0%

1

## Data Availability

The datasets used and/or analyzed during the current study are available from the corresponding author.
